# Predictors of mortality in critically ill patients with COVID-19 and diabetes

**DOI:** 10.1590/1414-431X2023e12728

**Published:** 2023-08-14

**Authors:** A.P.P. Lázaro, M.S. Zaranza, G.C. Meneses, N.L. Aragão, M.V.P. Freire, Á.R. Guimarães, A.M. Beliero, M.M.P. Dantas, L.C. Forte, A.M.C. Martins, E.F. Daher, P.L.M.M. Albuquerque, G.B. da Silva

**Affiliations:** 1 Programa de Pós-Graduação em Ciências Médicas, Departamento de Medicina Clínica, Curso de Medicina, Universidade Federal do Ceará, Fortaleza, CE, Brasil; 2 Programa de Pós-Graduação em Saúde Coletiva, Curso de Medicina, Centro de Ciências da Saúde, Universidade de Fortaleza, Fortaleza, CE, Brasil; 3 Centro de Ciências da Saúde, Curso de Medicina, Universidade de Fortaleza, Fortaleza, CE, Brasil; 4 Instituto José Frota (IJF) Hospital, Fortaleza, CE, Brasil; 5 Departamento de Análises Clínicas e Toxicológicas, Curso de Farmácia, Universidade Federal do Ceará, Fortaleza, CE, Brasil

**Keywords:** COVID-19, Diabetes, Biomarkers, Mortality, Dialysis

## Abstract

The COVID-19 pandemic has challenged the entire world, and patients with diabetes mellitus (DM) have been particularly affected. We aimed to evaluate predictors of mortality during the first 30 days of hospitalization in critically ill patients with COVID-19 and comorbid DM. This prospective study included 110 critically ill patients admitted with COVID-19 infection. Thirty-two (29%) patients had a previous diagnosis of DM. Clinical variables, laboratory tests, and vascular biomarkers, such as VCAM-1, syndecan-1, ICAM-1, angiopoietin-1, and angiopoeitin-2, were evaluated after intensive care unit (ICU) admission. A comparison was made between patients with and without DM. No difference in mortality was observed between the groups (48.7 *vs* 46.9%, P=0.861). In the multivariate Cox regression analysis, VCAM-1 levels at ICU admission (HR: 1 [1-1.001], P<0.006) were associated with death in patients with DM. Among patients with DM, advanced age (HR 1.063 [1.031-1.096], P<0.001), increased Ang-2/Ang-1 ratio (HR: 4.515 [1.803-11.308] P=0.001), and need for dialysis (HR: 3.489 [1.409-8.642], P=0.007) were independent predictors of death. Higher levels of VCAM-1 in patients with DM was better at predicting death of patients with severe COVID-19 and comorbid DM, and their cut-off values were useful for stratifying patients with a worse prognosis. Vascular biomarkers VCAM-1 and Ang-2/Ang-1 ratio were predictors of death in patients with severe COVID-19 and comorbid DM and those without DM. Additionally, kidney injury was associated with an increased risk of death.

## Introduction

Vascular involvement in COVID-19 is a trigger for a wide range of clinical manifestations, leading to severe acute respiratory syndrome. During the new coronavirus (SARS-CoV-2) pandemic, Brazil was one of the countries with the highest number of deaths (www.covid.saude.gov.br). Several predictive factors have been linked to COVID-19 severity and mortality, including increased inflammatory response, diabetes mellitus (DM), systemic arterial hypertension (SAH), and kidney injury (1).

An association exists between pro-inflammatory and pro-thrombotic factors in COVID-19, with consequent endothelial function impairment. Therefore, comorbidities associated with chronic endothelial dysfunction, such as DM, confer an increased susceptibility to severe disease (2,3).

According to previous studies, individuals with DM are more susceptible to COVID-19 infection. However, our understanding of disease progression in this group of patients remains unclear (4). Endothelial dysfunction is a hallmark of DM and is linked, in part, to the balance between oxidative stress and the nitric oxide synthase system (5). Oxidative stress can affect the expression of endothelial adhesion molecules since the signal transduction pathways for these molecules are associated with free radical generation (5).

The connection between COVID-19 and DM is multifactorial and bidirectional. Several risk factors found in patients with DM contribute to complications and death in patients with COVID-19. These factors include advanced age, pro-inflammatory status, hypercoagulability, and hyperglycemia and associated comorbidities (hypertension, cardiovascular disease, chronic kidney disease, and obesity). Treatment of severe COVID-19 involves the use of corticoids, which in turn negatively affects DM itself by raising glycemic levels through increased insulin resistance and reduced pancreatic beta cell function. Hyperglycemia leads to immune dysfunction, which decreases interleukin (IL) production in response to infection, reducing chemotaxis, phagocytic response, and polymorphonuclear leukocyte recruitment (1,6).

Relevant changes in the endothelium due to COVID-19 have enabled studies about new biomarkers, which could predict outcomes and result in better management of the patients. Vascular biomarkers have been classified into groups according to their function and action, such as endothelial inflammation (including vascular cell adhesion protein-1 (VCAM-1) and intracellular adhesion molecule-1 (ICAM-1)), thrombosis (including thrombomodulin, plasminogen activation inhibitor, and PAI-1), and glycocalyx (including syndecan-1) (7). ICAM-1 and VCAM-1 are markers of endothelial dysfunction that are elevated in patients with DM and correlate with glycated hemoglobin levels, indicating an association between glycemic control and expression of these molecules (8). In patients with DM, ICAM-1 and VCAM-1 are associated with markers of inflammation, such as C-reactive protein (CRP), IL-6, and tumor necrosis factor (TNF)-α. Endothelial dysfunction is associated with inflammation and has been shown to predict the risk of death in patients with DM (5).

Therefore, this study sought to investigate possible predictors of mortality during the first 30 days of hospitalization in critically-ill patients with COVID-19 with and without comorbid DM. These findings can provide strategies for early diagnosis, ensure rational use of resources, and encourage the development of new studies on better therapeutic approaches.

## Material and Methods

### Study design and selection of patients with COVID-19

This prospective study was conducted with 110 critically ill patients admitted to the intensive care unit (ICU) due to COVID-19 in a large tertiary hospital in the city of Fortaleza, Ceará state, northeastern Brazil, between June 2020 and June 2021. During this period, the first and second waves of the pandemic occurred in Brazil. The study included patients of both sexes who were aged ≥18 years, had a confirmed diagnosis of COVID-19 by RT-PCR, agreed to participate in the study, and provided written informed consent. Patients admitted for other causes than COVID-19 or who acquired COVID-19 during the hospital stay were excluded. Additionally, patients who died during the first 24 h of ICU admission and patients with incomplete information in their medical records were excluded.

Patients with DM were considered according to their self-reported previous diagnosis or their family, since almost all severe patients presented hyperglycemia on hospital admission. Patients with type 1 diabetes were not included. Primary care patient records were checked to confirm all diagnoses.

### Laboratory and clinical parameters of patients with COVID-19

The patients were followed throughout their ICU stay. The medical records were assessed for patient characteristics, such as symptoms, sociodemographic parameters, comorbidities, ICU length of stay, time between symptom onset and ICU admission, supportive care (vasopressor use, need for mechanical ventilation, and dialysis), and survival on Day 30 of hospitalization. The severity of disease in patients admitted to the ICU was estimated using the Simplified Acute Physiology Score 3 (SAPS3). SAPS3 is an international tool that uses data from patient admission to the ICU to assess the likelihood of death in hospital outcome (9). SAPS3, oxygenation index (the ratio of arterial oxygen partial pressure (PaO_2_ in mmHg) to fractional inspired oxygen (FiO_2_)), mortality, ICU length of stay, and all the ICU data were calculated using a cloud database program (EpimedTM System^®^) [https://www.epimedsolutions.com/sistema-epimed-monitor-uti/].

All recruited patients had blood samples collected on hospital admission. These samples were centrifuged (2000 *g*, room temperature, 15 min), aliquoted, and frozen at -80°C until the biomarkers analyses.

### Endothelial biomarker measurements

Endothelial biomarkers were quantified by ELISA assays, using isolated serum samples of the enrolled patients. Specific kits were acquired from R&D Systems^®^ (USA) for angiopoietin-1 (Ang-1) (cat# DY623), angiopoietin-2 (Ang-2) (cat# DY623), ICAM-1 (cat# DY279-05), and angiotensin converting enzyme-2 (ACE-2) (cat# DY4269). In the case of VCAM-1 and syndecan-1, kits from Abcam^®^ (USA) (ab47355 and ab47352) were used. The procedures were followed according to the manufacturers' recommendations.

### Statistical analysis

Categorical data are reported as absolute counts and percentages. The chi-squared test or Fisher's exact test was used to evaluate the differences in categorical data. Continuous data were analyzed, and data normality was determined using the Shapiro-Wilk test and evaluation of histograms and Q-Q plots. Data considered normally distributed are reported as means±SD, and non-normally distributed data are reported as median and interquartile range. Normally distributed data were compared using Student's *t*-test, whereas non-normally distributed data were compared using the Mann-Whitney test.

ROC curve analysis was performed to evaluate the capacity of endothelial biomarkers to predict death in diabetic patients, and the area under the ROC curve (AUC-ROC) with 95% confidence intervals were calculated. The various cut-offs from the ROC curve of each biomarker were determined using the higher Youden index (Youden Index = sensitivity + specificity - 1).

Moreover, the patients were stratified according to the cut-off values obtained previously from the ROC curve analysis, aiming to assess the prognostic value for death during the first 30 days of ICU stay using Kaplan-Meier analysis. The log-rank test was used to evaluate the statistical difference between the two groups (lower *vs* higher than the cut-off). Moreover, the unadjusted and adjusted Cox proportional hazards regression analyses were performed using quantitative levels of endothelial biomarkers and parameters previously associated with death (using P<0.05 as a condition). The collinearity of variables was assessed. For the multivariate model, the backward elimination method with likelihood ratio was used as a stepwise method. Briefly, all selected variables were evaluated in an initial model and then they were removed in a stepwise manner (removal criterion: P>0.10) until the final model, which contained predictors that contributed significantly to explain the event (death), was obtained. The data were analyzed using SPSS software for Macintosh, version 23 (IBM Corp., USA). A P-value <0.05 was considered statistically significant for all analytical tests.

### Ethics

This study was approved by the National Research Ethics Committee (CAAE number: 30579020.4.1001.0008). The patients were informed about the study purpose and, after accepting to participate, they signed a free and informed consent form before the evaluation. As some admitted patients required immediate ICU care and were unable to provide consent, a family member signed the form.

## Results

### Clinical and biomarker characteristics of patients with severe COVID-19 and comorbid DM

**Figure 1:**
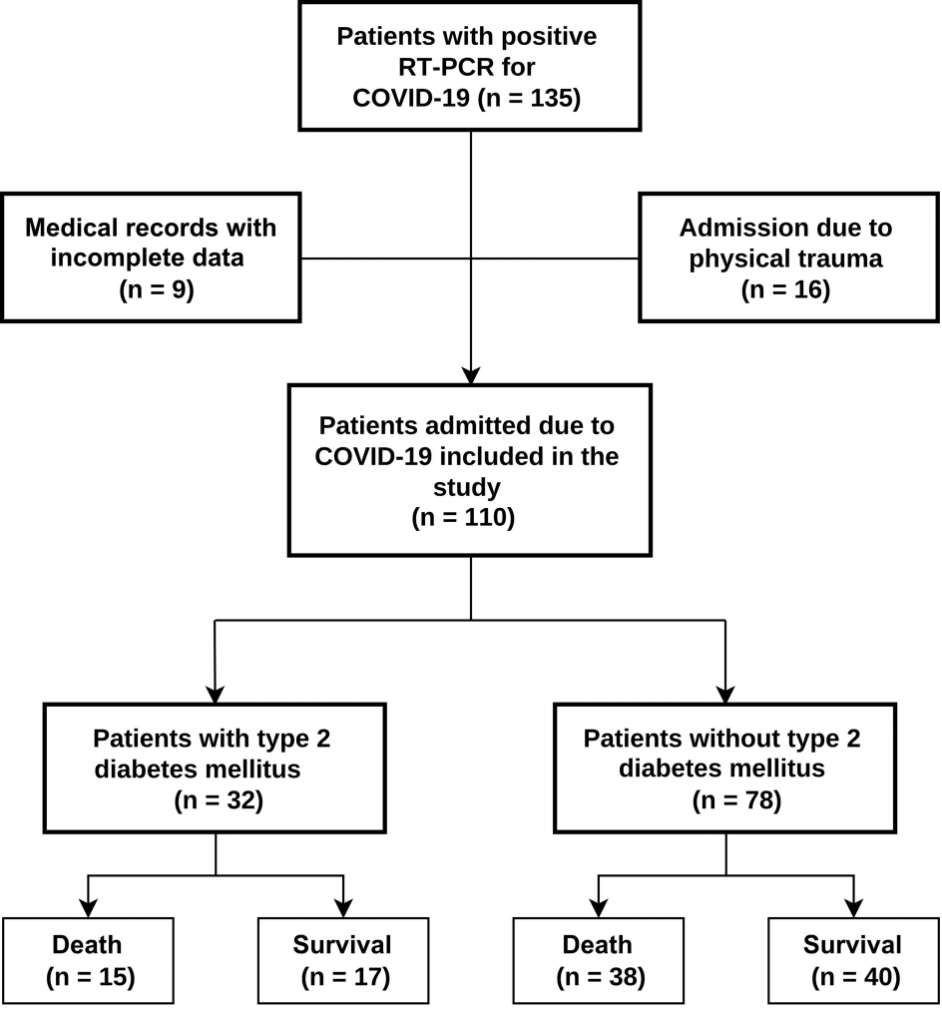
Flowchart of included COVID-19 patients.

A total of 110 patients with COVID-19 were enrolled in this study. Among these patients, 32 (29%) patients had a diagnosis of DM (Figure 1). Baseline epidemiologic, clinical, and laboratory characteristics were compared between patients with DM and those without DM (Table 1). Regarding the epidemiological profile, the DM group had more women (59.4 *vs* 32.1%; P=0.008) and older mean age (66 *vs* 56 years; P<0.001) than the group without DM. SAH was a more prevalent comorbidity in patients with DM (93.8 *vs* 30.8%; P<0.001). No differences in clinical parameters and complications, such as SAPS3 score, need for dialysis and respiratory support, and death, were observed between the groups. Furthermore, urea (P=0.006), creatinine (P=0.023), and CRP levels (P=0.046) were higher in patients with DM than those without DM.

**Table 1:** Comparison of epidemiologic, clinical, and laboratory characteristics between critically ill diabetic and non-diabetic COVID-19 patients.

Characteristics	Non-diabetic patients (n=78)	Diabetic patients (n=32)	P
Gender			0.008
Female	25 (32.1)	19 (59.4)	
Male	53 (67.9)	13 (40.6)	
Age (years)	56±19	66±8	<0.001
Obesity	23 (29.5)	12 (37.5)	0.413
Hypertension	24 (30.8)	30 (93.8)	<0.001
Alcoholism	8 (10.3)	0 (0)	0.102
Time between symptom onset and ICU admission (days)	9 (5-13)	11 (5-20)	0.314
Time of ICU stay (days)	9 (3-16)	11 (7-17)	0.254
SAPS3 score	58±18	61±16	0.539
Dialysis	28 (35.9)	16 (50)	0.17
Vasopressor use	33 (49.3)	17 (58.6)	0.399
Respiratory support			0.996
Invasive	44 (61.1)	19 (61.3)	
Non-invasive	23 (31.9)	10 (32.3)	
None	5 (6.9)	2 (6.5)	
Death	38 (48.7)	15 (46.9)	0.861
Laboratory and biomarker data			
Blood glucose (mg/dL)	126.5 (91-160.5)	156 (125-299)	0.004
VCAM-1 (ng/mL)	1281 (811-2289)	1542 (911-3350)	0.093
Syndecan (ng/mL)	161.3 (78.7-318)	190,5 (88.2-376.3)	0.624
ACE-2	2.05 (0.68-4.98)	1.52 (0.75-4.41)	0.785
ICAM-1 (ng/mL)	124 (74-172)	102 (81-159)	0.475
Ang-1 (ng/mL)	20.5 (11.36-29.09)	21.4 (9.88-33.77)	0.79
Ang-2 (ng/mL)	2.24 (1.31-4.53)	3.44 (1.64-7.04)	0.105
Hemoglobin (g/L)	11.4±2.4	10.9±2.7	0.284
Leukocytes (per mm^3^)	13439±5718	13266±5919	0.891
Lymphocytes (per mm^3^)	1199±809	1049±552	0.362
Platelet count (10^3^/mm^3^)	245096±114706	266793±119152	0.395
INR	1.07 (1.02-1.21)	1.09 (1.06-1.16)	0.384
aPTT	1.1 (1.02-1.33)	1.18 (1.06-1.33)	0.499
Serum urea (mg/dL)	44 (26-84)	67 (44-126)	0.006
Serum creatinine (mg/dL)	0.8 (0.6-1.3)	1.1 (0.8-2.4)	0.023
Serum potassium (mg/dL)	4.14±0.73	4.43±0.63	0.072
Serum sodium (mg/dL)	145±8	143±9	0.427
AST (U/L)	43 (31-70)	50 (34-63)	0.569
ALT (U/L)	45 (28-67)	39 (28-53)	0.409
LDH (U/L)	740 (540-897)	805 (621-1152)	0.208
Total bilirubin (mg/dL)	1 (0-1)	1 (0-1)	0.482
C-reactive protein (pg/mL)	126.6 (35-169,2)	169.95 (98.6-224.1)	0.046
D-dimer (ng/mL)	2 (1-4)	2 (1-4)	0.605

Quantitative data are reported as means±SD or median and interquartile range between parenthesis, according to the distribution of data. Qualitative data are reported as absolute count and percentages between parentheses. The chi-squared test or Fisher exact test was applied to qualitative data and Student's *t-*test or Mann-Whitney test was applied to quantitative data, according to normality. VCAM-1: vascular cell adhesion protein-1; ACE-2: angiotensin converting enzyme-2; ICAM-1: intracellular adhesion molecule-1; Ang-1: angiopoietin-1; Ang-2: angiopoietin-2; INR: international normalized ratio; aPPT; activated partial thromboplastin time; AST: aspartate transaminase; ALT: alanine transaminase; LDH: lactate dehydrogenase.

### Factors associated with death in patients with severe COVID-19 and comorbid DM

Epidemiological and clinical characteristics were compared between the outcomes of survival and death in patients with severe COVID-19 and comorbid DM (Table 2). The number of patients requiring dialysis was higher in the group that did not survive (80 *vs* 23.5%; P=0.001). No differences were observed in epidemiological and other clinical features, such as sex, age, prevalence of hypertension and obesity, SAPS3 score, and need for respiratory support.

**Table 2:** Comparison between epidemiologic characteristics, clinical features, laboratory and biomarker results according to survival in severe COVID-19 patients with diabetes mellitus.

Diabetic COVID-19 patients	Survival (n=17)	Non-survival (n=15)	P
Gender			0.513
Female	11 (64.7)	8 (53.3)	
Male	6 (35.3)	7 (46.7)	
Age (years)	68±8	64±8	0.234
Obesity	5 (29.4)	7 (46.7)	0.314
Hypertension	16 (94.1)	14 (93.3)	1
Alcoholism	0 (0)	0 (0)	-
Time between symptom onset and ICU admission (days)	10 (2-17)	11 (5-34)	0.412
Time of ICU stay (days)	13 (7-18)	8 (7-16)	0.595
SAPS3	55±12	66±18	0.067
Dialysis	4 (23.5)	12 (80)	0.001
Vasopressor use	6 (42.9)	11 (73.3)	0.096
Respiratory Support			0.087
Invasive	7 (43.8)	12 (80)	
Non-invasive	7 (43.8)	3 (20)	
None	2 (12.5)	0 (0)	
Laboratory and biomarker data			
Blood glucose (mg/dL)	181.5 (122-299)	153 (144-253)	0.905
VCAM-1 (ng/mL)	934 (659-1421)	3569 (1822-6214)	<0.001
Syndecan (ng/mL)	93.4 (52.8-172.1)	288 (196.8-747)	0.002
ACE-2	1.48 (0.69-2.44)	1.56 (0.8-5.06)	0.737
ICAM-1 (ng/mL)	88 (73-142)	105 (92-168)	0.123
Ang-1 (ng/mL)	31.37 (20.23-48.63)	12.4 (8-29.49)	0.014
Ang-2 (ng/mL)	2.23 (1.24-5.42)	5.53 (1.83-7.21)	0.295
Hemoglobin (g/L)	11.1±2.4	10.6±3	0.576
Leukocytes (per mm^3^)	13741±5145	12756±6813	0.662
Lymphocytes (per mm^3^)	1175±679	913±350	0.203
Platelet count (10^3^/mm^3^)	306067±125356	224714±99847	0.065
INR	1.1 (1.06-1.18)	1.09 (1.03-1.16)	0.936
aPTT	1.15 (0.95-1.24)	1.27 (1.06-1.43)	0.149
Serum urea (mg/dL)	45 (30-77)	123 (67-139)	0.001
Serum creatinine (mg/dL)	0.9 (0.7-1.1)	2.25 (0.9-3.4)	0.012
Serum potassium (mg/dL)	4.25±0.61	4.61±0.61	0.133
Serum sodium (mg/dL)	144±7	143±11	0.853
AST (U/L)	48 (34-57)	56 (33-78)	0.437
ALT (U/L)	39 (28-44)	43 (26-66)	0.437
LDH (U/L)	729 (551-1025)	855 (665-1152)	0.487
Total bilirubin (mg/dL)	1 (0-1)	1 (0-1)	0.361
C-reactive protein (pg/mL)	215.85 (131.45-260.3)	153.35 (62.15-195.2)	0.16
D-dimer (ng/mL)	2 (1-3)	2 (1-7)	0.805

Quantitative data are reported as means±SD or median and interquartile range between parenthesis, according to the distribution of data. Qualitative data are reported as absolute count and percentages between parentheses. The chi-squared test or Fisher exact test was applied to qualitative data and Student's *t*-test or Mann-Whitney test was applied to quantitative data, according to normality. VCAM-1: vascular cell adhesion protein-1; ACE-2: angiotensin converting enzyme-2; ICAM-1: intracellular adhesion molecule-1; Ang-1: angiopoietin-1; Ang-2: angiopoietin-2; INR: international normalized ratio; aPPT: activated partial thromboplastin time; AST: aspartate transaminase; ALT: alanine transaminase; LDH: lactate dehydrogenase.

The laboratory data of patients with severe COVID-19 and comorbid DM were analyzed and compared between the survival and non-survival groups (Table 2). When compared to the survival group, patients with DM who died had higher levels of VCAM-1 (3569 [1822-6214] *vs* 934 [659-1421]; P<0.001), syndecan-1 (288 [196.8-747] *vs* 93.4 [52.8-172.1]; P=0.002), creatinine (2.25 [0.9-3.4] *vs* 0.9 [0.7-1.1]; P=0.012), and urea (123 [67-139] *vs* 45 [30-77]; P=0.001) but a lower Ang-1 level (12.4 [8-29.49] *vs* 31.37 [20.23-48.63]; P=0.014). See Supplementary Table S1 for data of non-diabetic patients with severe COVID-19.

**Figure 2:**
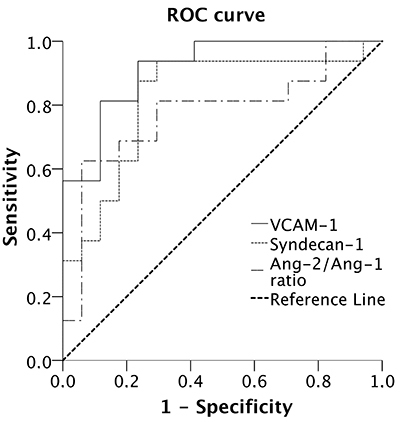
Receiver operating characteristic (ROC) curve analysis of intensive care unit admission and endothelial biomarkers levels in diabetic COVID-19 patients. VCAM-1: vascular cell adhesion protein-1 Ang-1: angiopoietin-1; Ang-2: angiopoietin-2.

The performance of endothelial biomarkers in predicting death in patients with severe COVID-19 and comorbid DM was evaluated using ROC curve analysis. VCAM-1 level had higher AUC-ROC (0.91; P<0.001) (Figure 2 and Table 3). The VCAM-1 cut-off, which was 1.441 ng/mL, had sensitivity and specificity of 93 and 76.5%, respectively. The syndecan-1 cut-off, which was 140.5 ng/mL, had sensitivity and specificity of 93 and 70%, respectively, whereas the Ang-2/Ang-1 ratio cut-off of 0.411 had sensitivity and specificity of 60 and 94%, respectively (Table 3).

**Table 3:** Performance of endothelial biomarkers in predicting death of diabetic patients with severe COVID-19.

	Cut-off	Sensitivity (%)	Specificity (%)	AUC-ROC (95%CI)	P
VCAM-1 (ng/mL)	1441	93	76.5	0.91 (0.812-1.0)	<0.001
Syndecan-1 (ng/mL)	140.5	93	70	0.820 (0.664-0.976)	0.002
Ang-2/Ang-1 ratio	0.411	60	94	0.765 (0.588-0.941)	0.011

VCAM-1: vascular cell adhesion protein-1; Ang-1: angiopoietin-1; Ang-2: angiopoietin-2; AUC-ROC: area under the receiver operating characteristics curve.

Cox regression analysis was applied to evaluate the independent significance of endothelial biomarkers for death outcome in patients with severe COVID-19 and comorbid DM. In the group of patients with DM, increased VCAM-1 levels at ICU admission remained associated with death due to COVID-19 in the multivariate model (HR: 1 [1-1.001], P<0.006). For patients without DM, older age (HR 1.063 [1.031-1.096], P<0.001), increased Ang-2/Ang-1 ratio at ICU admission (HR: 4.515 [1.803-11.308] P=0.001), and need for dialysis (HR: 3.489 [1.409-8.642]; P=0.007) were independent predictors of death (Table 4).

**Table 4:** COX regression analysis with adjusted risk factors for death in COVID-19 patients with or without type-2 diabetes mellitus in the study.

	Non-diabetic COVID-19 patients				Diabetic COVID-19 patients			
	Initial model		Final model		Initial model		Final model	
	Hazard ratio (95%CI)	P	Hazard ratio (95%CI)	P	Hazard ratio (95%CI)	P	Hazard ratio (95%CI)	P
SAPS3	0.999(0.971-1.027)	0.92	-	-	1.017(0.964-1.073)	0.537	-	-
Age, years	1.065(1.029-1.102)	<0.001	1.063(1.031-1.096)	<0.001	0.924(0.825-1.035)	0.172	-	-
Gender, male	1.172(0.468-2.932)	0.735	-	-	1.304(0.32-5.308)	0.711	-	-
VCAM-1 (ng/mL)	1(0.999-1)	0.439	-	-	1(1-1.001)	0.051	1(1-1.001)	0.006
Syndecan-1 (ng/mL)	1.001(0.999-1.003)	0.338	-	-	0.998(0.995-1)	0.083	0.999(0.997-1)	0.136
Ang-2/Ang-1 ratio	4.792(1.749-13.126)	0.002	4.515(1.803-11.308)	0.001	1.15(0.382-3.461)	0.804	-	-
Dialysis	2.967(1.09-8.075)	0.033	3.489(1.409-8.642)	0.007	3.387(0.268-42.797)	0.346	-	-
Serum creatinine (mg/dL)	0.823(0.552-1.229)	0.341	0.748(0.53-1.056)	0.099	0.915(0.565-1.482)	0.717	-	-

SAPS3: Simplified Acute Physiology Score 3; VCAM-1: vascular cell adhesion protein-1; Ang-1: angiopoietin-1; Ang-2: angiopoietin-2.

**Figure 3:**
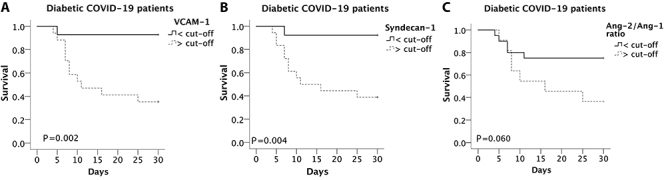
Survival evaluation according to endothelial biomarker cut-off values of vascular cell adhesion protein-1 (VCAM-1), syndecan-1, and angiopoietin (Ang)-2/Ang-1 ratio at admission to the intensive care unit from receiver operating characteristic (ROC) curve analysis in diabetic COVID-19 patients.

Survival curves were constructed for comparisons between groups according to cut-off values of each endothelial biomarker. Patients with DM with VCAM-1 values above the cut-off values (>1441 ng/mL) and syndecan-1 above the cut-off (>140.5 ng/mL) at ICU admission died more quickly than those who had lower values than the cut-off values (for VCAM-1: mean=18.1 days [95%CI: 14.9-21.3] *vs* 25.3 [23.1-27.6], P=0.002), (for syndecan-1: 19.1 days [16.2-22.0] *vs* 25.5 [23.1-27.9], P=0.004) (Figure 3).

## Discussion

Studies on new endothelial biomarkers have emerged as a proposal to identify early complications related to severe COVID-19. DM is a clinical condition with several macro- and microvascular complications, which negatively affect the COVID-19 patient overall survival. Correlating DM with COVID-19 is challenging, given that even in patients with DM, SARS-CoV-2 may cause transient hyperglycemia, which may interfere in the management and prognosis of both groups of patients (1).

Patients with severe COVID-19 and comorbid DM have shown peculiar clinical and epidemiological features. In the current study, these individuals were older and more associated with SAH and exacerbated inflammatory response than the patients without DM, as evidenced by the higher CRP levels. Accordingly, some studies have reported similar results in a similar population (10).

Several studies have investigated the effect of DM on COVID-19 severity. In a meta-analysis of 76 studies involving 31,067 patients with COVID-19, patients with DM had a significantly higher risk of more severe disease progression (RR: 2.38) and mortality (RR: 2.1) (11). A retrospective cohort of 178 patients with DM hospitalized with COVID-19 in the United States found that these patients had a 59% higher risk of needing ICU admission, a 97% higher risk of needing mechanical ventilation, and a two-fold higher risk of death after the model was adjusted for age, sex, ethnicity, and BMI (12).

The mechanism of kidney dysfunction induced by COVID-19 infection is multifactorial, involving both the direct invasion of the virus and indirect mechanisms, such as acute tubular necrosis, immune system dysregulation, hyper-coagulopathy, and collapsing glomerulopathy (13). Acute kidney injury (AKI) was present in >20% of patients with severe COVID-19 in studies conducted in China, the United States, and Italy (14). Among the prior comorbidities of patients with COVID-19, the morbidity most often related to the progression of kidney function decline was DM (15). Similar to this study, other studies have reported that need for dialysis during hospital stay was associated with death (16,17). In a multicenter cohort study of patients with COVID-19 undergoing hemodialysis, severe courses of the disease and mortality were substantially increased compared to the general population affected by COVID-19 (18). In our study, patients who were undergoing dialysis prior to hospital admission were excluded, as the chronic inflammation related with chronic kidney disease in renal replacement treatment may interfere with the clinical and laboratory parameters.

In the DM group, the biomarker VCAM-1 stood out as a marker of poor prognosis. Mechanistically, the disruption of the endothelial barrier by the SARS-COV-2 spike protein is mediated by changes in the surface expression of ICAM-1 and VCAM-1. Plasma levels of these adhesion molecules are often elevated in these patients, especially in the more critical ones (19). Accordingly, some studies have demonstrated that circulating levels of VCAM-1 reflect endothelial activation and damage (20). Another study reported that VCAM-1 levels were higher in patients with COVID-19 and moderate-to-severe respiratory failure who did not survive (21). A recent study demonstrated that patients with COVID-19 have higher levels of VCAM-1 than controls with the same clinical presentation but without SARS-CoV-2 infection, supporting the concept that COVID-19-associated pneumonia specifically affects the endothelium (22). Notably, we highlight that the DM group had a significantly higher number of patients that required dialysis. However, we know these parameters are closely interconnected, as endothelial dysfunction has been reported to underlie the severe evolution of COVID-19. Therefore, VCAM-1 levels may predict early AKI and allow efficient measures to be taken to prevent disease progression and its complications in this group of patients.

The glycocalyx consists of a gel-like layer on the surface of healthy endothelium that prevents contact between blood cells and vascular endothelium. Syndecan-1 (SYN-1) is a product of glycocalyx degradation after endothelial injury and inflammation. The breakdown of the barrier function occurs secondary to the loss of both glycocalyx and endothelial cell-cell adhesion complexes, leading to the entry of plasma proteins and the increase in water permeability, similar to sepsis and other severe conditions (7). In our study, SYN-1 levels were correlated with a high risk of death among patients with DM. Previous data suggest that high SYN-1 levels are a potential marker for COVID-19 progression or severity, as they are associated with more severe endothelial damage and inflammatory reactions. Otherwise, high SYN-1 level has been associated with increased thrombomodulin, IL-6, and TNF-α levels (23).

In our study, the Ang2/Ang1 ratio was shown to be a predictor of severity among patients without DM. Angiopoietins are part of a group of vascular growth factors that play a role in angiogenesis. Ang-1 acts by reorganizing endothelial cells and promoting barrier function. Ang-2 essentially acts as an antagonist of Ang-1, and its increased expression is related to endothelial activation (24). Increased Ang-2 level is related to endothelial activation and increased risk of ICU admission in patients admitted with COVID-19 (25).

No difference was observed regarding the outcome of death between the DM and non-DM groups. This can be explained due to the scenario in which both groups were inserted, with severe conditions and with similar SAPS3 scores. Therefore, the presence of multiple underlying comorbidities in this inpatient profile may explain the high death rate in both groups. Although DM was not independently associated with mortality in patients with COVID-19, this and other comorbidities should not be analyzed separately. An example of this situation is the presence of both DM and SAH, which often coexist and can act synergistically to promote adverse clinical events (26). In a Chinese study of hospitalized patients with COVID 19, 153 patients with DM were more likely to have a history of SAH than 153 patients without DM who were matched for sex and age (56.9 *vs* 28.8%) (26). Other associated clinical conditions may have influenced this outcome, since only patients admitted to the ICU who were known to have more severe conditions were included in this study. Therefore, the scenario of severity and ICU hospitalization, the associated comorbidities, and the clinical picture must be analyzed to determine the outcome.

At this point of the pandemic, this study could reach different results due to the emergence of new variants of the virus and the availability of new therapies and vaccines. Vaccine effectiveness studies have shown that COVID-19 vaccination can reduce severe illness and affect the use of hospital resources (27,28). However, studies with biomarkers that correlate with mortality are necessary because vaccine-resistant variants may emerge in the future.

### Strengths and limitations

This study highlighted important clinical predictors of mortality in patients with COVID-19. A major limitation is related to the fact that the study was conducted in a single center with a small number of patients. Additionally, some information, especially patients' anthropometric data, were missing.

### Conclusion

Endothelial activation, characterized by elevated serum levels of VCAM-1, was associated with death in patients with severe COVID-19 and comorbid DM. Clear evidence of how biomarker levels can change the course of disease according to the severity of COVID-19 infection exists and can be used as a complement in clinical practice to guide patient screening, treatment, and admission to the ICU for a differentiated follow-up, thus improving prognosis and minimizing hospitalization and mortality rates.

## Supplementary material

Click to view [pdf].

## References

[1] Magdy Beshbishy A, Oti VB, Hussein DE, Rehan IF, Adeyemi OS, Rivero-Perez N (2021). Factors behind the higher COVID-19 risk in diabetes: a critical review. Front Public Health.

[2] Brandão SCS, Godoi ETAM, Ramos JOX, de Melo LMMP, Dompieri LT, Brindeiro DF, Sarinho ESC (2020). Papel do endotélio na COVID-19 grave. Arq Bras Cardiol.

[3] Jin Y, Ji W, Yang H, Chen S, Zhang W, Duan G (2020). Endothelial activation and dysfunction in COVID-19: from basic mechanisms to potential therapeutic approaches. Signal Transduct Target Ther.

[4] Mostaza JM, García-Iglesias F, González-Alegre T, Blanco F, Varas M, Hernández-Blanco C (2020). Clinical course and prognostic factors of COVID-19 infection in an elderly hospitalized population. Arch Gerontol Geriatr.

[5] Endemann DH, Schiffrin EL (2004). Nitric oxide, oxidative excess, and vascular complications of diabetes mellitus. Curr Hypertens Rep.

[6] Landstra CP, de Koning EJP (2021). COVID-19 and Diabetes: Understanding the Interrelationship and Risks for a Severe Course. Front Endocrinol (Lausanne).

[7] Flaumenhaft R, Enjyoji K, Schmaier AA (2022). Vasculopathy in COVID-19. Blood.

[8] Wen Y, Skidmore JC, Porter-Turner MM, Rea CA, Khokher MA, Singh BM (2002). Relationship of glycation, antioxidant status and oxidative stress to vascular endothelial damage in diabetes. Diabetes Obes Metab.

[9] Moreno RP, Metnitz PGH, Almeida E, Jordan B, Bauer P, Campos RA (2005). SAPS 3 - From evaluation of the patient to evaluation of the intensive care unit. Part 2: Development of a prognostic model for hospital mortality at ICU admission. Intensive Care Med.

[10] Yan Y, Yang Y, Wang F, Ren H, Zhang S, Shi X (2020). Clinical characteristics and outcomes of patients with severe covid-19 with diabetes. BMJ Open Diabetes Res Care.

[11] Shang L, Shao M, Guo Q, Shi J, Zhao Y, Xiaokereti J (2020). Diabetes mellitus is associated with severe infection and mortality in patients with COVID-19: a systematic review and meta-analysis. Arch Med Res.

[12] Seiglie J, Platt J, Cromer SJ, Bunda B, Foulkes AS, Bassett IV (2020). Diabetes as a risk factor for poor early outcomes in patients hospitalized with COVID-19. Diabetes Care.

[13] Geetha D, Kronbichler A, Rutter M, Bajpai D, Menez S, Weissenbacher A (2022). Impact of the COVID-19 pandemic on the kidney community: lessons learned and future directions. Nat Rev Nephrol.

[14] Sardu C, Gambardella J, Morelli MB, Wang X, Marfella R, Santulli G (2020). Hypertension, thrombosis, kidney failure, and diabetes: is COVID-19 an endothelial disease? A comprehensive evaluation of clinical and basic evidence. J Clin Med.

[15] Cravedi P, Mothi SS, Azzi Y, Haverly M, Farouk SS, Pérez-Sáez MJ (2020). COVID-19 and kidney transplantation: results from the TANGO International Transplant Consortium. Am J Transplant.

[16] Hirsch JS, Ng JH, Ross DW, Sharma P, Shah HH, Barnett RL (2020). Acute kidney injury in patients hospitalized with COVID-19. Kidney Int.

[17] Wang X, Fang X, Cai Z, Wu X, Gao X, Min J, Wang F (2020). Comorbid chronic diseases and acute organ injuries are strongly correlated with disease severity and mortality among COVID-19 patients: a systemic review and meta-analysis. Research (Wash DC).

[18] Seidel M, Hölzer B, Appel H, Babel N, Westhoff TH, COVID Dialysis Working Group (2020). Impact of renal disease and comorbidities on mortality in hemodialysis patients with COVID-19: a multicenter experience from Germany. J Nephrol.

[19] Perico L, Benigni A, Casiraghi F, Ng LFP, Renia L, Remuzzi G (2021). Immunity, endothelial injury and complement-induced coagulopathy in COVID-19. Nat Rev Nephrol.

[20] Libby P, Lüscher T (2020). COVID-19 is, in the end, an endothelial disease. Eur Heart J.

[21] Lampsas S, Tsaplaris P, Pantelidis P, Oikonomou E, Marinos G, Charalambous G (2022). The role of endothelial related circulating biomarkers in COVID-19. A systematic review and meta-analysis. Curr Med Chem.

[22] Dalla Sega FV, Fortini F, Spadaro S, Ronzoni L, Zucchetti O, Manfrini M (2021). Time course of endothelial dysfunction markers and mortality in COVID-19 patients: A pilot study. Clin Transl Med.

[23] Zhang D, Li L, Chen Y, Ma J, Yang Y, Aodeng S (2021). Syndecan-1, an indicator of endothelial glycocalyx degradation, predicts outcome of patients admitted to an ICU with COVID-19. Mol Med.

[24] Fiedler U, Augustin HG (2006). Angiopoietins: a link between angiogenesis and inflammation. Trends Immunol.

[25] Smadja DM, Guerin CL, Chocron R, Yatim N, Boussier J, Gendron N (2020). Angiopoietin-2 as a marker of endothelial activation is a good predictor factor for intensive care unit admission of COVID-19 patients. Angiogenesis.

[26] Shi Q, Zhang X, Jiang F, Zhang X, Hu N, Bimu C (2020). Clinical characteristics and risk factors for mortality of COVID-19 patients with diabetes in Wuhan, China: a two-center, retrospective study. Diabetes Care.

[27] Tenforde MW, Self WH, Adams K, Gaglani M, Ginde AA, McNeal T (2021). Association between mRNA vaccination and COVID-19 hospitalization and disease severity. Jama.

[28] Stepanova M, Lam B, Younossi E, Felix S, Ziayee M, Price J (2022). The impact of variants and vaccination on the mortality and resource utilization of hospitalized patients with COVID-19. BMC Infect Dis.

